# A randomized controlled trial of an intervention delivered by mobile phone text message to increase the acceptability of effective contraception among young women in Palestine

**DOI:** 10.1186/s13063-019-3297-4

**Published:** 2019-04-23

**Authors:** Ona L. McCarthy, Hanadi Zghayyer, Amina Stavridis, Samia Adada, Irrfan Ahamed, Baptiste Leurent, Phil Edwards, Melissa Palmer, Caroline Free

**Affiliations:** 1 Department of Population Health, Faculty of Epidemiology and Population Health, London School of Hygiene & Tropical Medicine, Keppel Street, London, WC1E 7HT UK; 2 Palestinian Family Planning & Protection Association, Industrial Zone, Wadi Al-Joze, Jerusalem, Palestine; 3 International Planned Parenthood Federation, Arab World Regional Office, 2 Place Virgile, Notre Dame, 1082 Tunis, Tunisia; 4 Department of Medical Statistics, Faculty of Epidemiology and Population Health, London School of Hygiene & Tropical Medicine, Keppel Street, London, WC1E 7HT UK

**Keywords:** Palestine, Contraception, mHealth, Family planning, Young adults

## Abstract

**Background:**

Research has shown that mobile phone contraceptive behavioral interventions can increase knowledge and use of contraception, but other studies have failed to demonstrate a beneficial effect. The objective of this trial was to estimate the effect of a contraceptive behavioral intervention delivered by mobile phone text message on young Palestinian women’s attitudes towards effective contraception.

**Methods:**

We conducted a randomized controlled trial among women aged 18–24 years living in the West Bank, who were not using an effective method of contraception. The intervention group received zero to three messages per day (113 messages for female-not married and 120 messages for female-married) for 120 days. The control group received 16 messages over 120 days about trial participation. The primary outcome was acceptability of at least one method of effective contraception at 4 months. Secondary outcomes were use of effective contraception at 4 months and any use during the study, acceptability of individual methods, service uptake, unintended pregnancy and abortion. Process outcomes included knowledge, perceived norms, personal agency and intention. All outcomes were self-reported. We analyzed the outcomes using logistic and linear regression.

**Results:**

A total of 578 participants were enrolled and 464 (80%) completed follow up at 4 months. Intervention group participants were more likely to find at least one method of effective contraception acceptable (31% in the intervention group versus 17% in the control group, adjusted OR 2.34, 95% CI 1.48–3.68, *p* < 0.001). They had a higher mean knowledge score, were more likely to find the intrauterine device, injection, implant and patch acceptable, to agree that their friends would use an effective method and to intend to use an effective method, compared to participants in the control group. While in the direction of intervention benefit, there were no differences between the groups in the use of effective contraception at 4 months and any use during the study, pill acceptability, service uptake, unintended pregnancy and induced abortion.

**Conclusions:**

The intervention can improve attitudes, knowledge-perceived norms and intention to use effective contraception among young women in Palestine. Research is needed to evaluate the efficacy of the intervention for contraceptive behavioral outcomes in Palestine.

**Trial registration:**

ClinicalTrials.gov, NCT02905461. Registered on 14 September 2016.

World Health Organization Trial Registration Data Set: http://apps.who.int/trialsearch/Trial2.aspx?TrialID=NCT02905461

**Electronic supplementary material:**

The online version of this article (10.1186/s13063-019-3297-4) contains supplementary material, which is available to authorized users.

## Background

Unintended pregnancy continues to be a global health problem [1]. Women with an unmet need of modern contraception account for an estimated 84% of all unintended pregnancies in developing regions [2]. Satisfying unmet need of modern contraception is essential in avoiding unintended pregnancies, and identifying the barriers that prevent people from using contraceptive methods can help achieve this [3, 4].

Sexual and reproductive health in the State of Palestine (the West Bank, East Jerusalem and the Gaza Strip, hereafter referred to as “Palestine”) has been negatively affected by the conflict [5, 6]. It is estimated that 38% of pregnancies are unintended [7], with the unmet need of contraception peaking at 15% among women aged 20–24 years [8]. While the adolescent fertility rate has decreased substantially over the past 20 years, the current adolescent fertility rate of 48 per 1000 women aged 15–19 years remains higher than in most other countries in the region [8, 9]. The Palestinian Family Survey 2010 found that among married women not using contraception and not reporting wanting to have a child, the main reasons given for not using contraception were fear of side effects, the inconvenience of the contraceptive methods and their husband’s opposition [10, 11].

Mobile phones are commonly used to deliver health behavioral support [12–18]. In Palestine, delivering contraceptive support by mobile phone may be an advantageous mode by which to reach people in the substantial area that is underserved with regard to sexual and reproductive health services [19]. There is some evidence from trials that interventions delivered by mobile phone can improve contraceptive use [20–22] and knowledge [20, 23–25]; however, other trials have not found a beneficial effect [26–30].

The London School of Hygiene and Tropical Medicine (LSHTM) and the Palestinian Family Planning and Protection Association (PFPPA) developed a contraceptive behavioral intervention in Palestine, which is delivered by mobile phone [31]. This paper reports the results of the evaluation of the intervention.

## Methods

The methods are summarized in this section. Detailed methods are published in the trial protocol [32] and the statistical analysis plan [33].

### Aim, study design and participants

This was a parallel group, individually randomized trial with a 1:1 allocation ratio. The aim was to assess the effect of the intervention on attitudes towards the non-permanent effective contraceptive methods [34–36] available in Palestine (oral contraceptive pills (OCs), intrauterine devices (IUDs), injectables, implants and the patch). Women were eligible to take part if they were between 18 and 24 years of age, did not report using an effective method of contraception, owned a personal mobile phone, lived in the West Bank and could read Arabic. The trial was promoted through PFPPA’s service delivery points and outreach sites, the PFPPA website and the distribution of trial promotional material via flyers and social media sites. We recruited participants through PFPPA’s service delivery points and outreach sites in Jerusalem, Bethlehem, Halhoul and Ramallah.

### Intervention and control

The intervention was informed by the integrated behavioral model [37] and was sent by mobile phone text message. It was tailored according to marital status, resulting in two sets of intervention messages: (1) female-married and (2) female-not married. Most of the messages in the two sets overlap, with minor tailoring so that the messages are relevant to marital status (marital status was used as a proxy for sexual activity because in this context it was thought inappropriate to ask about sexual activity if not married). Participants allocated to the intervention group received zero to three messages per day (113 messages for female-not married and 120 messages for female-married) for 120 days.

The intervention messages provided information about contraception, targeted beliefs identified in the development phase that influence contraceptive use (e.g. misconceptions about the side effects and health risks of contraception, belief that non-hormonal methods are better because they are not harmful to health) and aimed to support young women in believing that they can influence their reproductive health. The intervention contained the following behavior change methods, adapted for delivery by mobile phone [38]: belief selection, facilitation, anticipated regret, guided practice, verbal persuasion, tailoring, cultural similarity, arguments, shifting perspective and goal setting.

Participants allocated to the control group received 16 control messages about trial participation over 120 days. Details on the development of the intervention can be found in the intervention development publication [31] and the trial protocol [32].

### Allocation and intervention delivery

The online trial database and randomization system were used to generate the allocation sequence, and randomization occurred immediately after the baseline data were submitted by the clinic research staff. The system sent the Palestinian texting platform the allocation, preferred time slot for message delivery, mobile phone number and marital status.

### Protecting against bias

Participants would have been aware of the allocation after they started receiving the messages. Allocation was masked from the research staff collecting outcome data unless the participant revealed it to them. Treatment allocation was masked from the researchers who analyzed the data.

### Outcomes

#### Primary outcome

The primary outcome was the proportion of participants reporting that at least one method of effective contraception was acceptable at 4 months post randomization. The acceptability of each method was measured by the following stems: Using the [method] … causes infertility, … causes unwanted side effects, … is easy, … is a good way to prevent pregnancy and I would recommend the [method] to a friend. The IUD and implant included an additional stem: The [method] insertion would not be a problem for me. The response options for each stem were: strongly disagree, disagree, not sure, agree, strongly agree and I do not know what the [method] is. A method was acceptable if participants reported agree or strongly agree for all stems except for … causes infertility and … causes unwanted side effects, for which disagree or strongly disagree indicated acceptability [32].

#### Secondary outcomes

Secondary outcomes were the use of effective contraception at 4 months and any use during the study, the acceptability of individual methods and service uptake, unintended pregnancy and abortion.

##### Process outcomes

The process outcomes were knowledge of effective contraception; perceived norms and personal agency in relation to using and communicating with partners about contraception and intention to use effective contraception and intervention dose received.

### Data collection

All outcomes were self-reported. At baseline, we collected personal and demographic data and the primary outcome data via self-completed paper questionnaire. We collected data on all outcomes at 4-month follow up. Staff, masked to treatment allocation, collected the follow-up data verbally by telephone.

### Sample size

The trial was powered to detect a 15% absolute increase in the acceptability (corresponding to an odds ratio (OR) of 1.86) of effective contraception in the intervention group compared to the control group. A sample of 454 participants would provide 90% power to detect a 15% absolute increase in acceptability, at the 5% significance level, assuming 50% acceptability in the control group. We allowed for 20% loss to follow up and aimed to randomize 570 participants.

### Statistical analysis

The trial protocol was accepted for publication on 14 September 2017 [32] and the detailed statistical analysis plan was publicly released before conducting the data analysis, on 7 November 2017 [33]. Analyses were conducted according to randomized group and only participants with complete outcome data were included in the principal analysis. All statistical tests were two-sided and considered significant at the 5% level. The analysis was conducted using Stata 15. Unmasking occurred on 6 February 2018, after the masked data were analyzed as outlined within the analysis plan.

#### Loss to follow up and missing data

We used the chi-squared test to investigate whether loss to follow up differed by trial arm. We used logistic regression to compare baseline characteristics of participants who completed follow up with participants who did not. We investigated whether predictors of loss to follow up differed by trial arm by testing for an interaction.

#### Principal analysis

##### Analysis of the primary outcome

We used logistic regression to compare the proportion of participants in each trial arm who reported that at least one method of contraception was acceptable. We report the crude and adjusted OR with the 95% confidence interval (CI) and *p* value. We adjusted the primary analysis regression for prespecified baseline covariates [32, 33].

##### Analysis of the secondary outcomes

The analysis of the secondary outcomes was similar to the analysis of the primary outcome, although in analysis of the acceptability of the individual methods, the acceptability of that method at baseline replaced the acceptability of at least one method at baseline as a covariate.

##### Analysis of the process outcomes

The process outcomes of perceived norms, personal agency and intention each comprised ordinal scales. Each scale was analyzed individually using ordered logistic regression. For knowledge, we used linear regression to assess the difference in the mean scores between the groups. To quantify the “dose” of the intervention that the intervention participants received, we analyzed the number of messages that participants reported to have read (all, most, some, none) and whether they stopped the messages, along with our monitoring data.

#### Additional analyses

##### Sensitivity analyses

We conducted two sensitivity analyses to account for missing data. In the first, we considered that all participants who were lost to follow up did not find at least one method acceptable. In the second, we adjusted for the main baseline predictors of missingness. Both sensitivity analyses were adjusted for the baseline covariates as aforementioned.

##### Subgroup analysis

We conducted an exploratory subgroup analysis for the primary outcome with prespecified subgroups [33]. Within the subgroups, we assessed the heterogeneity of the estimated treatment effect with a test for interaction [39, 40]. We estimated ORs with 95% CIs for each subgroup.

##### Contamination

We report the proportion of control group participants who reported that they read another participant’s messages and the proportion of intervention participants that reported that their messages were read by another participant.

##### Report of physical violence

We report the proportion of participants in each group that reported experiencing physical violence during the study.

## Results

### Recruitment, randomization, exclusions

Between 8 December 2016 and 22 July 2017, 586 randomizations were performed by the system. During the trial follow-up we discovered that four of the participants enrolled were randomized twice. The two participants who were allocated to the same group on both randomizations were kept in the trial, using the baseline data from their first record (the system allowed only one follow-up record). We excluded the two participants who were allocated to different groups from the analysis.

**Fig. 1:**
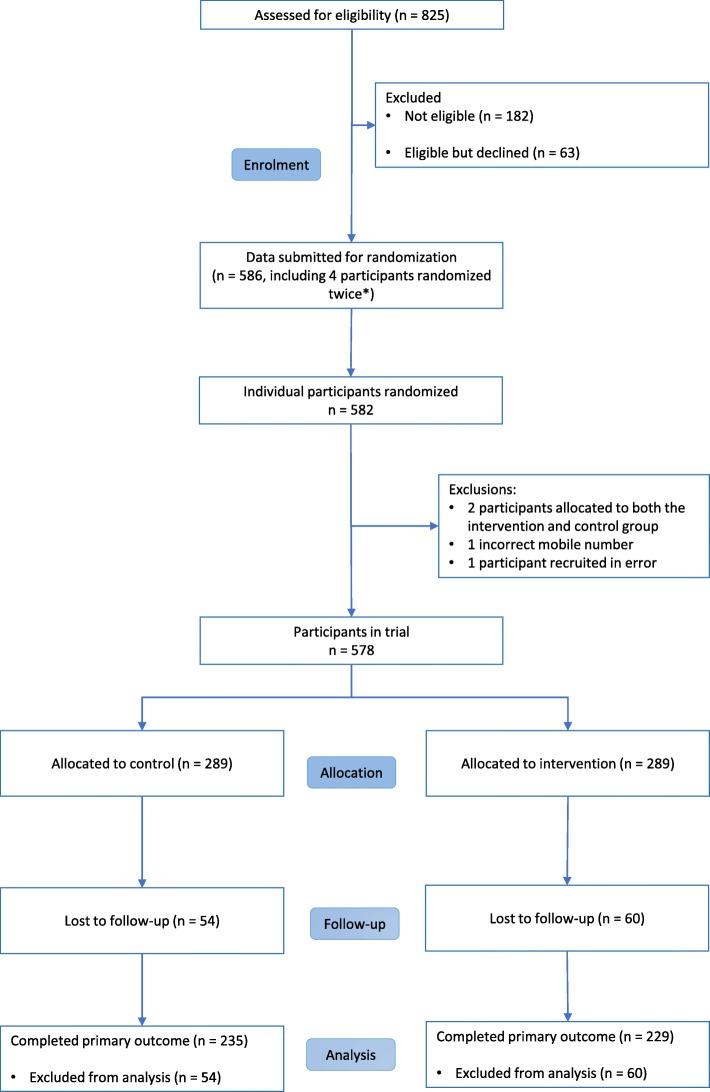
Consolidated standards of reporting trials (CONSORT) diagram

In addition, one record was excluded because the participant’s incorrect mobile number was entered onto the database (the correct record and number was kept) and another record was excluded because the participant was recruited in error (the participant was using an IUD and was therefore ineligible). This resulted in 578 participants being included in the trial (Fig. 1).

There were 289 participants allocated to the intervention group and 289 to the control group. No participants withdrew from the trial after allocation.

### Baseline characteristics

Baseline characteristics of trial participants are reported in Table 1. Mean age was 21 years, and 71% (409/578) were aged 20–24 years: 60% of participants were not married (259/573) and only 8% (47/578) found at least one method of effective contraception acceptable. The characteristics were largely similar between the two groups; however, almost twice as many participants in the control group reported that at least one method of effective contraception was acceptable at baseline compared to the intervention group (10.38% in the control versus 5.88% in the intervention group; see “Discussion”).

**Table 1:** Baseline characteristics

	Control *N* = 289, %(*n*)	Intervention *N* = 289, %(*n*)	All participants *N* = 578, %(*n*)
Age, years			
mean (sd)	21.4 (1.77)	21.2 (1.75)	21.3 (1.76)
18–19	25.9 (75)	32.5 (94)	29.2 (169)
20–24	74.0. (214)	67.5 (195)	70.8 (409)
Marital status			
married	40.5 (117)	38.8 (112)	39.6 (229)
not-married	59.5 (172)	61.3 (177)	60.4 (349)
Number of children			
0	72.3 (209)	79.6 (230)	75 (439)
1	16.3 (47)	10.7 (31)	13.5 (78)
2 or more	11.4 (33)	9.7 (28)	10.6 (61)
Residence			
city	46.7 (135)	47.8 (138)	47.2 (273)
village	48.1 (139)	46.7 (135)	47.4 (274)
camp	4.2 (12)	4.8 (14)	4.5 (26)
Bedouin community	1 (3)	0.7 (2)	0.9 (5)
Occupation			
school^a^	1.7 (5)	0.4 (1)	1.0 (6)
university	48.4 (140)	52.6 (152)	44.6 (258)
working	5.2 (15)	3.5 (10)	4.3 (25)
training	14.9 (43)	15.9 (46)	15.2 (88)
parent	22.5 (65)	20.1 (58)	20.6 (119)
not working	5.9 (17)	6.6 (19)	6.2 (36)
university and working	0.4 (1)	0.4 (1)	0.4 (2)
university and parent	0.7 (2)	–	0.4 (2)
school and parent	0.4 (1)	0.4 (1)	0.4 (2)
working, training and parent	–	0.4 (1)	0.2 (1)
Highest level of education completed			
primary	0.7 (2)	0.7 (2)	0.7 (4)
secondary	22.8 (66)	21.1 (61)	22 (127)
university	66.1 (191)	66.4 (192)	66.3 (383)
technical	10.4 (30)	11.8 (34)	11.1 (64)
Current pregnancy intention (“Do you want a pregnancy now?”)			
yes	16.3 (47)	20.1 (58)	18.2 (105)
no	25.6 (74)	24.6 (71)	25.1 (145)
unsure	4.2 (12)	1.4 (4)	2.8 (16)
not married^b^	53 (156)	53 (156)	53 (312)
Baseline method			
none	39.5 (114)	41.5 (120)	40.5 (234)
male condom	0.7 (2)	0.7 (2)	0.7 (4)
not married^b^	53.6 (155)	54.3 (157)	53 (312)
calendar	1.0 (3)	0.4 (1)	0.7 (4)
LAM	3.1 (9)	1.4 (4)	2.3 (13)
withdrawal	2.1 (6)	1.4 (4)	1.7 (10)
other	–	0.4 (1)	0.2 (1)
At least one effective method is acceptable			
yes	10.4 (30)	5.9 (17)	8.1 (47)
no	89.6 (259)	94.1 (272)	91.9 (531)
Pill acceptability			
yes	3.8 (11)	3.1 (9)	3.5 (20)
no	96.2 (278)	96.9 (280)	96.5 (558)
IUD acceptability			
yes	4.5 (13)	1.7 (5)	3.1 (18)
no	95.5 (276)	98.3 (284)	96.9 (560)
Injection acceptability			
yes	1.4 (4)	1.4 (4)	1.4 (8)
no	98.6 (285)	98.6 (285)	98.6 (570)
Implant acceptability			
yes	3.1 (9)	1.7 (5)	2.4 (14)
no	96.9 (280)	98.3 (284)	97.6 (564)
Patch acceptability			
yes	0.7 (2)	0.4 (1)	0.5 (3)
no	99.3 (287)	99.7 (288)	99.5 (575)

*LAM* lactational amenorrhea method, *IUD* intrauterine device

^a^School is pre-university education

^b^The response “not married” was used as a proxy for sexual activity

### Loss to follow up

A total of 464 participants (80%) completed the trial follow up for the primary outcome (control, *n* = 235; intervention, *n* = 229) (Fig. 1). Retention did not differ between the groups (81% in the control and 79% in the intervention group, Pearson’s chi squared test *p* = 0.53). The main predictor of retention was completion of university at enrolment (OR 1.80, 95% CI 1.18–2.73, *p* = 0.01). The effect of this predictor of retention did not differ by group (interaction test *p* = 0.78). Detailed characteristics of follow-up completers and non-completers are reported in Additional file 1.

### Outcomes

In the intervention group, 31% (71/229) reported that at least one method of contraception was acceptable compared to 17% (40/235) in the control group (Table 2). Participants in the intervention group had 2.34 times the odds of finding at least one method of effective contraception acceptable compared to participants in the control group (adjusted OR 2.34, 95% CI 1.48–3.68, *p* < 0.001; crude OR 2.19, 95% CI 1.41–3.40, *p* < 0.001; absolute risk difference = 14%, 95% CI 0.06-0.22, *p* < 0.001). The odds of participants having an IUD, injection, implant, patch or long-acting reversible contraception (LARC) method were greater in the intervention group compared to the control group and were statistically significant (Table 3). The odds of using effective contraception was also greater in the intervention group; however, this could likely have occurred by chance (Table 2). Participants in the intervention group had a higher mean knowledge score, were more likely to agree that their friends would use an effective contraceptive method and were more likely to respond that they intend to use an effective method compared to participants in the control group (Table 3).

**Table 2:** Primary and secondary outcomes

	Control *N* = 235, % (*n*)	Intervention *N* = 229, % (*n*)	Adjusted OR (95% CI)	*p* value
At least one effective method is acceptable^a^	17 (40)	31 (71)	2.34 (1.48–3.68); absolute risk difference = 14% (0.06–0.22)	< 0.001
Use of effective contraception^a^	8.5 (20)	8.7 (20)	1.42 (0.66–3.07)	0.37
Pill acceptability^b^	4.7 (11)	6.1 (14)	1.39 (0.61–3.16)	0.44
IUD acceptability^b^	6.4 (15)	14 (32)	2.76 (1.41–5.40)	0.003
Injection acceptability^b^	1.7 (4)	5.7 (13)	3.16 (0.99–10.08)	0.05
Implant acceptability^b^	5.5 (13)	11.8 (27)	2.46 (1.19–5.07)	0.02
Patch acceptability^b^	2.6 (6)	10 (23)	4.17 (1.63–10.64)	0.003
LARC acceptability^b^	11.9 (28)	23.1 (53)	2.49 (1.48–4.18)	0.001
Any effective contraceptive use during the 4 months^a^	8.1 (19)	10 (23)	1.95 (0.90–4.25)	0.09
Service uptake^a^ (attended a service one or more times)	37 (87)	42.8 (98)	1.38 (0.94–2.04)	0.10
Unintended pregnancy^a^	3.1 (9/289)	2.4 (7/289)	0.75 (0.27–2.10)	0.59
Induced abortion^a^	2.6 (6)	1.3 (3)	0.47 (0.11–1.95)	0.30

*IUD* intrauterine device, *LARC* long-acting reversible contraception

^a^Adjusted for pregnancy intention, age, number of children, education level and acceptability at baseline

^b^Adjusted for pregnancy intention, age, number of children, education level and the corresponding method acceptability at baseline

**Table 3:** Process outcomes

	Control *N* = 235, % (*n*)	Intervention *N* = 229, % (*n*)	Proportional OR^a^ (95% CI), *p* value
Knowledge of effective contraception	Mean = 2.13 (sd = 1.42)	Mean = 2.63 (sd = 1.66)	0.50 ^b^ (0.22–0.78), 0.001
My friends would use the pill, IUD, injection or implant if they wanted to prevent pregnancy			
strongly disagree	0.9 (2)	0.4 (1)	1.46 (1.00–2.13), 0.05
disagree	9.4 (22)	5.7 (13)	
not sure	21.7 (51)	17 (39)	
agree	62.1 (146)	70.7 (162)	
strongly agree	6 (14)	6.1 (14)	
My friends would talk to their husband about contraception if they wanted to prevent a pregnancy			
strongly disagree	–	–	0.92 (0.63–1.34), 0.66
disagree	2.6 (6)	2.6 (6)	
not sure	18.3 (43)	19.7 (45)	
agree	66.4 (156)	65.9 (151)	
strongly agree	12.8 (30)	11.8 (27)	
If you wanted to use the pill, IUD, injection or implant, how easy would it be for you to use it?			
very difficult	0.9 (2)	1.3 (3)	1.26 (0.89–1.78), 0.19
difficult	9.8 (23)	8.7 (20)	
not sure	48.9 (115)	41.1 (94)	
easy	35.3 (83)	46.3 (106)	
very easy	5.1 (12)	2.6 (6)	
If you wanted to talk to your husband about contraception, how easy would it be for you to talk to him?			
very difficult	1.3 (3)	1.8 (4)	0.83 (0.59–1.17), 0.29
difficult	7.2 (17)	9.6 (22)	
not sure	17.9 (42)	17.5 (40)	
easy	51.5 (121)	52.8 (121)	
very easy	22.1 (52)	18.3 (42)	
If you wanted to use the pill, IUD, injection or implant, how certain are you that you could use it?			
very certain I could not	1.3 (3)	0.9 (2)	1.19 (0.84–1.68), 0.33
certain I could not	4.7 (11)	4.4 (10)	
not sure	43.4 (102)	39.7 (91)	
certain I could	43.8 (103)	47.2 (108)	
very certain I could	6.8 (16)	7.9 (18)	
If you wanted to talk to your husband about contraception, how certain are you that you could talk to him?			
very certain I could not	–	–	1.05 (0.73–1.50), 0.80
certain I could not	2.6 (6)	3.5 (8)	
not sure	18.7 (44)	11.8 (27)	
certain I could	53.6 (126)	63.3 (145)	
very certain I could	25.1 (59)	21.4 (49)	
I intend to use the pill, IUD, injection, implant or patch			
strongly disagree	2.1 (5)	2.6 (6)	1.85 (1.29–2.65), 0.001
disagree	13.6 (32)	4.4 (10)	
not sure	24.7 (58)	18.3 (42)	
agree	51.1 (120)	63.8 (146)	
strongly agree	8.5 (20)	10.9 (25)	
Number of messages read			
all		62.9 (144)	
most		21.8 (50)	
some		11.4 (26)	
none		3.9 (9)	
Proportion of intervention participants that stopped the intervention		3.9 (9)	

*IUD* intrauterine device

^a^Estimated from ordered logistic regression

^b^Mean difference

### Additional trial data

Of the control participants, 17% (39/235) said that they read the messages of someone else in the study; 17% (40/229) of intervention participants said that someone else in the study read their messages. In the intervention group, 0.87% (2/229) reported that they experienced physical violence during the study versus 2.13% (5/235) in the control group (Fisher’s exact test *p* = 0.45). Most intervention participants who answered the question '*Did anything good or bad happend as a result of receiving the messages*?' (193/229) said that they benefitted from the messages, mainly from the increase in information and awareness about the contraceptive methods. No participants reported any serious negative events that happened during the study.

### Sensitivity analyses

The effect of the intervention on the primary outcome observed in the principal analysis (adjusted OR 2.34, 95% CI 1.48–3.68, *p* < 0.001) moved slightly towards the null when we considered participants who were lost to follow up did not find at least one method of effective contraception acceptable (OR 2.13, 95% CI 1.37–3.30, *p* = 0.001).

The strongest baseline predictor of retention was having completed university. This was already adjusted for in the primary analysis and the sensitivity analysis model was the same as the primary analysis.

### Subgroup analysis

**Fig. 2:**
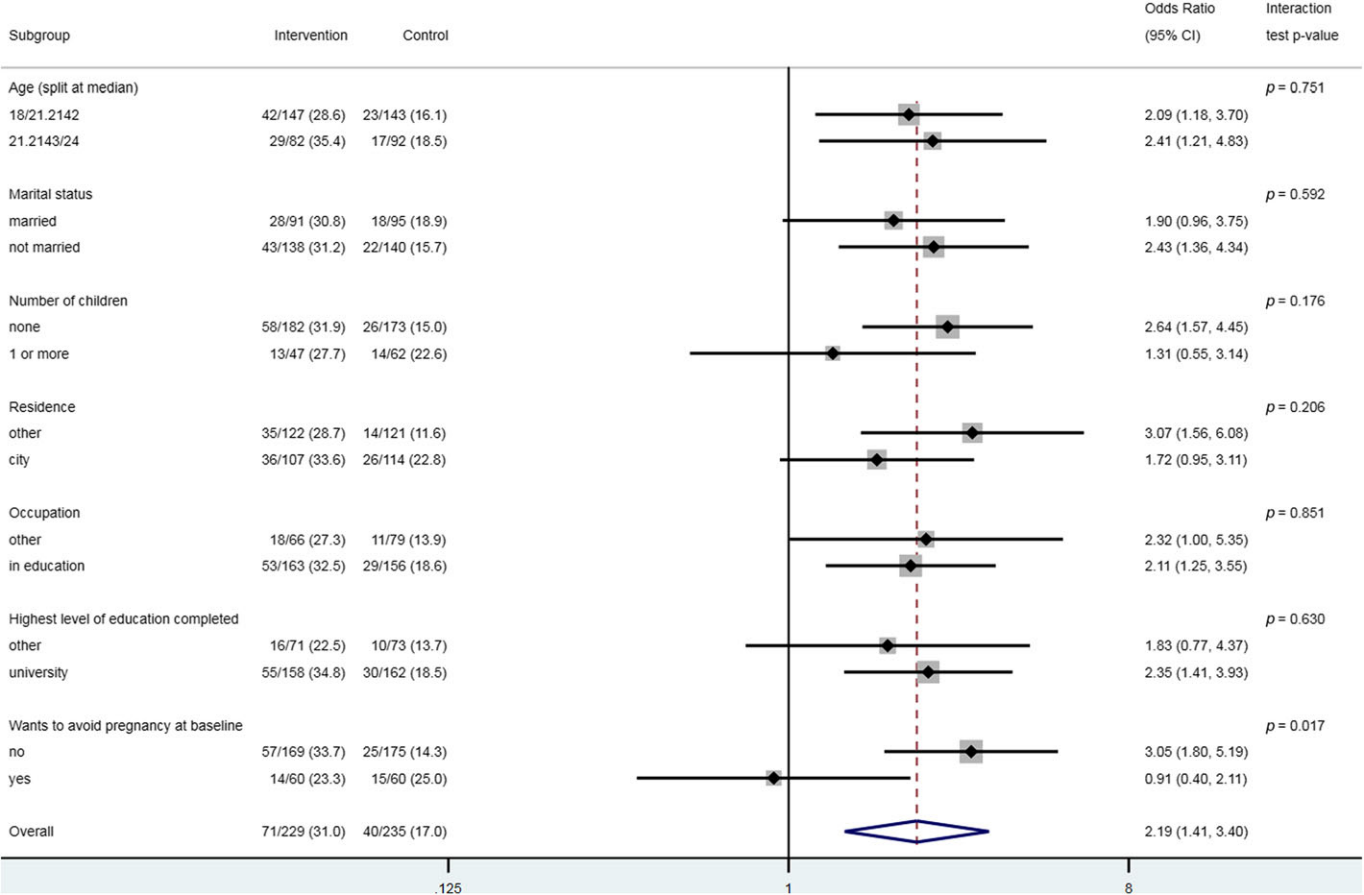
Primary outcome by prespecified subgroups

The intervention was less effective among participants who wanted to avoid pregnancy at baseline compared to participants who did not (interaction test *p* = 0.02) (Fig. 2).

### Intervention delivery

After the trial commenced, there were two technical problems with the local platform that resulted in participants not being sent the full intervention. Based on the data available from the local platform, 40% of intervention participants were sent 90% or more of the intervention messages and 8% of intervention participants were sent less than 70% of the messages.

## Discussion

### Main results

The results of this trial demonstrated that the intervention improved young women’s attitudes towards effective contraception in Palestine. The trial results also suggest that the intervention moderately improves knowledge about effective contraception, perceived norms about friends using effective contraception and intention to use effective contraception. The subgroup analysis suggests that that the intervention may be less effective among women who want to avoid pregnancy at baseline.

### Comparisons with other research

We identified 11 trials evaluating individual-level interventions delivered by mobile phone to improve contraceptive-related outcomes [20–30], using search terms from a previous Cochrane review [41]. Of the five trials where knowledge of contraception was an outcome [20, 23–25, 30], four showed a beneficial effect of the intervention compared to the control [20, 23–25]. Both trials where knowledge of contraception was the primary outcome showed a beneficial effect [24, 25]. All trials included a use-related outcome, however, only three showed a beneficial effect [20–22]. The results of this Palestine trial are in line with research that has found mobile phone interventions can increase contraceptive knowledge [20, 23–25]. Previous research has demonstrated that interventions delivered by mobile phone can improve contraceptive use [20–22]. This current trial did not find evidence of a difference in use between the groups, although this study did not have enough statistical power for this outcome. This is the first trial that we are aware of that has shown that a contraceptive behavioral intervention delivered by mobile phone messaging can increase intention to use effective contraception. A similar intervention was evaluated by a trial in Tajikistan [42] and in Bolivia with young people (manuscript in preparation). A post hoc change from baseline to follow-up analysis of all participants in Tajikistan demonstrated a large increase in acceptability [42].

### Strengths and limitations

An important strength of this trial is that we recruited to target and achieved greater than 80% follow-up for the primary outcome. Our trial database and randomization system concealed the allocation sequence and achieved well-balanced groups overall. There was, however, some imbalance in acceptability at baseline, but this was adjusted for in the primary analysis and had little effect on the results when not controlled for. The sensitivity analysis confirmed that our results were robust to different missing data assumptions.

The main limitation of the trial is that the whole intervention was not delivered to all participants due to technical problems with the local platform, so our result can only tell us the effect of partial receipt of the intervention. Another limitation is that all outcomes were self-reported, which meant that they are more likely to be biased than if they were objectively measured. For example, the self-reported primary outcome collected by telephone by research staff may have meant that participants were more likely to report positive attitudes at follow up compared to baseline where they provided data by paper questionnaire. In addition, the scoring of the primary outcome measure may have led to an underestimation of the true acceptability of effective contraception. In scoring the measure, we thought it better to avoid false positives; for a method of effective contraception to be acceptable, participants had to choose “agree” or “strongly agree” to the positively worded stems and “disagree” or “strongly disagree” to the negatively worded stems. In other words, for participants to score “acceptable” they must have unequivocally thought the method acceptable. It is possible that an individual could believe that a method had unwanted side effects but would still use it because they felt that the benefit of using it outweighed the risks (that is, an unintended pregnancy is more unwanted than the perceived side effect). While this does not have implications for the effect of the intervention relative to the control, it means that in the sample overall, the true acceptability may have been higher at baseline and at follow up.

Most participants were university educated. The inclusion of participants from a wider range of socio-economic backgrounds would have improved the generalizability of the results. Although sexual activity before marriage is highly stigmatized in Palestine, it is possible that some non-married participants were sexually active. In this case, while it is the best available measure of sexual activity in this context, marital status would not have been a reliable proxy. While we did not record why participants assessed for eligibility were ineligible or were eligible but declined to participate, 91% of eligible women assessed (582/643) were randomized.

### Meaning and implications of the findings

The intervention effect estimated in this trial is likely to be conservative due to the moderate level of potential contamination and the fact that an estimated 60% of intervention participants were not sent the full intervention. Further analysis of the trial data and of the data on the local platform could evaluate the dose of the intervention on the outcomes.

The finding that the intervention may be less effective among women who want to avoid pregnancy at baseline, could relate to exposure to information about contraception. Women who want to avoid pregnancy at baseline may already have formed positive attitudes towards the effective methods. Indeed, the level of acceptability at baseline was higher among participants wanting to avoid pregnancy compared to those who wanted to become pregnant, were unsure or were not married (12% versus 7%), reducing the potential for improvement.

Due to the nature of the intervention, participants would have been aware of their treatment allocation. It is possible that this awareness, along with the greater number of intervention messages compared to control messages could have contributed to the improvement of attitudes towards effective contraception observed in the intervention group rather than the content of the intervention itself.

Most trial participants were recruited through PFPPA’s services. This group may have had a higher level of acceptability of effective contraception, compared to people who were not in any way connected to or aware of family planning services. However, the low baseline acceptability in the trial sample (8%) suggests that acceptability is low in general in the West Bank. Acceptability among intervention participants remained relatively low at 31%. Further analysis of the individual scales that comprise the primary outcome measure and qualitative work could help clarify why the intervention did not improve acceptability to a greater extent.

Although not powered to detect differences in contraceptive behavioral outcomes, the effect estimates of the intervention for these outcomes (use of effective contraception, unintended pregnancy and induced abortion), while not statistically significant, were all in the direction of intervention benefit. A larger trial powered for these outcomes would provide a more precise estimate.

It is noteworthy that, although not statistically significant, the odds of finding the contraceptive pill acceptable in the intervention group relative to the control group were lowest out of all the methods. Further analysis of the baseline data showed that a larger proportion of participants at baseline knew what the pill was compared to the other methods. Because there was greater awareness of the pill in the study population at the outset, views on the method may have been less amenable to the influence of the intervention. Participants may have been less likely to have negative preconceived ideas about the other methods as there was less awareness of them. Further research could explore attitudes towards the pill versus other methods in the Palestinian context.

The results indicate that implementation of the intervention in Palestine could improve young women’s attitudes towards effective contraceptive methods. If the intervention could be delivered at low cost (e.g. though a mobile phone app rather than through mobile phone text message), its implementation would be justified and research would be required to understand how the implementation would work in a non-trial context. Implementation of the intervention delivered by text message, however, would require evidence that the intervention can improve behavioral outcomes, due to cost of sending text messages. If the intervention is made available, the local platform will need regular monitoring and maintenance to ensure that the intervention is delivered as intended.

## Conclusions

This trial demonstrated that the intervention more than doubled the odds of finding at least one method of effective contraception acceptable. This result along with the lack of evidence that it is associated with any harm, supports the implementation of the intervention in Palestine. Future research is needed to evaluate the efficacy of the intervention on use of effective contraception and unintended pregnancy in Palestine and to determine how to enable successful implementation.

## Additional file

Additional file 1: Baseline characteristics by follow-up. (DOCX 25 kb)

## Abbreviations

IUD: Intrauterine device
LARC: Long-acting reversible contraception
LSHTM: London School of Hygiene and Tropical Medicine
PFPPA: Palestinian Family Planning and Protection Association
