# Supplementary material for: A randomized controlled trial of an intervention delivered by mobile phone text message to increase the acceptability of effective contraception among young women in Palestine
Source: Trials. 2019 Apr 23;20:228. doi: 10.1186/s13063-019-3297-4 (PMC6477750; doi:10.1186/s13063-019-3297-4)
Supplement: Supplementary file 1 — Baseline characteristics by follow-up. (DOCX 25 kb) [file 13063_2019_3297_MOESM1_ESM.docx]

|  | | **Primary outcome completers** | | **Primary outcome non-completers** | | | **All participants** | | |
| --- | --- | --- | --- | --- | --- | --- | --- | --- | --- |
|  |  | **Control**  N = 235  % (n) | **Intervention**  N = 229  % (n) | | **Control**  N = 54  % (n) | **Intervention**  N = 60  % (n) | | **Control**  N = 289  % (n) | **Intervention**  N = 289  % (n) |
| Age | mean [sd] | 21.38 [1.73] | 21.18 [1.70] | | 21.29 [1.97] | 21.18 [1.93] | | 21.36 [1.77] | 21.18 [1.75] |
|  | 18-19 | 24.68 (58) | 32.75 (75) | | 31.48 (17) | 31.67 (19) | | 25.95 (75) | 32.53 (94) |
|  | 20-24 | 75.32 (177) | 67.25 (154) | | 68.52 (37) | 68.33 (41) | | 74.05 (214) | 67.47 (195) |
| Marital status | married | 40.43 (95) | 39.74 (91) | | 40.74 (22) | 35.00 (21) | | 40.48 (117) | 38.75 (112) |
|  | not-married | 59.57 (140) | 60.26 (138) | | 59.26 (32) | 65.00 (39) | | 59.52 (172) | 61.25 (177) |
| Number of children | 0 | 73.62 (173) | 79.48 (182) | | 66.67 (36) | 80.00 (48) | | 72.32 (209) | 79.58 (230) |
|  | 1 | 13.62 (32) | 10.92 (25) | | 27.78 (15) | 10.00 (6) | | 16.26 (47) | 10.73 (31) |
|  | 2 or more | 12.77 (30) | 9.61 (22) | | 5.56 (3) | 10.00 (6) | | 11.42 (33) | 9.69 (28) |
| Residence | city | 48.51 (114) | 46.72 (107) | | 38.89 (21) | 51.67 (31) | | 46.71 (135) | 47.75 (138) |
|  | village | 47.23 (111) | 48.1 (112) | | 51.85 (28) | 38.33 (23) | | 48.10 (139) | 46.71 (135) |
|  | camp | 3.40 (8) | 3.93 (9) | | 7.41 (4) | 8.33 (5) | | 4.15 (12) | 4.84 (14) |
|  | Bedouin community | 0.85 (2) | 0.44 (1) | | 1.85 (1) | 1.67 (1) | | 1.04 (3) | 0.69 (2) |
| Occupation | school | 2.13 (5) | 0.44 (1) | | - | - | | 1.73 (5) | 0.35 (1) |
|  | university | 50.21 (118) | 55.02 (126) | | 40.74 (22) | 43.33 (26) | | 48.44 (140) | 52.60 (152) |
|  | working | 4.26 (10) | 3.06 (7) | | 9.26 (5) | 5.00 (3) | | 5.19 (15) | 3.46 (10) |
|  | training | 12.34 (29) | 14.85 (34) | | 25.93 (14) | 20.00 (12) | | 14.88 (43) | 15.92 (46) |
|  | parent | 22.8 (54) | 19.65 (45) | | 20.37 (11) | 21.67 (13) | | 22.49 (65) | 20.07 (58) |
|  | not working | 6.38 (15) | 6.11 (14) | | 3.70 (2) | 8.33 (5) | | 5.88 (17) | 6.57 (19) |
|  | university & working | 0.43 (1) | 0.44 (1) | | - | - | | 0.35 (1) | 0.35 (1) |
|  | university & parent | 0.85 (2) | - | | - | - | | 0.69 (2) | - |
|  | school & parent | 0.43 (1) | - | | - | 1.67 (1) | | 0.35 (1) | 0.35 (1) |
|  | working, training & parent | - | 0.44 (1) | | - | - | | - | 0.35 (1) |
| Highest level of education completed | primary | 0.43 (1) | 0.87 (2) | | 1.85 (1) | - | | 0.69 (2) | 0.69 (2) |
|  | secondary | 21.70 (51) | 19.21 (44) | | 27.78 (15) | 28.33 (17) | | 22.84 (66) | 21.11 (61) |
|  | university | 68.94 (162) | 69.00 (158) | | 53.70 (29) | 56.67 (34) | | 66.09 (191) | 66.44 (192) |
|  | technical | 8.94 (21) | 10.92 (25) | | 16.67 (9) | 15.00 (9) | | 10.38 (30) | 11.76 (34) |
| Current pregnancy intention  (‘*Do you want a pregnancy now?)* | yes | 16.60 (39) | 20.52 (47) | | 14.81 (8) | 18.33 (11) | | 16.26 (47) | 20.07 (58) |
|  | no | 25.53 (60) | 26.20 (60) | | 25.93 (14) | 18.33 (11) | | 25.61 (74) | 24.57 (71) |
|  | unsure | 4.26 (10) | 1.75 (4) | | 3.70 (2) | - | | 4.15 (12) | 1.38 (4) |
|  | not married ^a^ | 53.62 (126) | 51.53 (118) | | 55.56 (30) | 63.33 (38) | | 53.98 (156) | 53.98 (156) |
| Baseline method | none | 41.28 (97) | 43.23 (99) | | 31.48 (17) | 35.00 (21) | | 39.45 (114) | 41.52 (120) |
|  | male condom | 0.85 (2) | 0.87 (2) | | - | - | | 0.69 (2) | 0.69 (2) |
|  | not married ^a^ | 52.34 (123) | 52.84 (121) | | 59.26 (32) | 60.00 (36) | | 53.63 (155) | 54.33 (157) |
|  | calendar | 0.85 (2) | 0.44 (1) | | 1.85 (1) | - | | 1.04 (3) | 0.35 (1) |
|  | LAM ^b^ | 2.55 (6) | 1.31 (3) | | 5.56 (3) | 1.67 (1) | | 3.11 (9) | 1.38 (4) |
|  | withdrawal | 1.70 (4) | 0.87 (2) | | 1.85 (1) | 3.33 (2) | | 2.08 (6) | 1.38 (4) |
|  | none-withdrawal | 0.43 (1) | - | | - | - | | - | - |
|  | other | - | 0.44 (1) | | - | - | | - | 0.35 (1) |
| At least one effective method is acceptable | yes | 9.36 (22) | 5.24 (12) | | 14.81 (8) | 8.33 (5) | | 10.38 (30) | 5.88 (17) |
|  | no | 90.64 (213) | 94.76 (217) | | 85.19 (46) | 91.67 (55) | | 89.62 (259) | 94.12 (272) |
| Pill acceptability | yes | 3.40 (8) | 2.62 (6) | | 5.56 (3) | 5.00 (3) | | 3.81 (11) | 3.11 (9) |
|  | no | 96.60 (227) | 97.38 (223) | | 94.44 (51) | 95.00 (57) | | 96.19 (278) | 96.89 (280) |
| IUD ^c^ acceptability | yes | 4.26 (10) | 0.87 (2) | | 5.56 (3) | 5.00 (3) | | 4.50 (13) | 1.73 (5) |
|  | no | 95.74 (225) | 99.13 (227) | | 94.44 (51) | 95.00 (57) | | 95.50 (276) | 98.27 (284) |
| Injection acceptability | yes | 1.28 (3) | 1.31 (3) | | 1.85 (1) | 1.67 (1) | | 1.38 (4) | 1.38 (4) |
|  | no | 98.72 (232) | 98.69 (226) | | 98.15 (53) | 98.33 (59) | | 98.62 (285) | 98.62 (285) |
| Implant acceptability | yes | 2.55 (6) | 1.31 (3) | | 5.56 (3) | 3.33 (2) | | 3.11 (9) | 1.73 (5) |
|  | no | 97.45 (229) | 98.6 (226) | | 94.44 (51) | 96.67 (58) | | 96.89 (280) | 98.27 (284) |
| Patch acceptability | yes | 0.85 (2) | 0.44 (1) | | - | - | | 0.69 (2) | 0.35 (1) |
|  | no | 99.15 (233) | 99.56 (228) | | 100 (54) | 100 (60) | | 99.31 (287) | 99.65 (288) |

^a^ the response ‘not married’ was used as a proxy for sexual activity

^b^ LAM Lactational amenorrhea method

^c^ IUD Intrauterine device
